# Protective effects of dill fruit against acute acetic acid‐induced ulcerative colitis by reducing inflammation and oxidative stress in rats

**DOI:** 10.14814/phy2.70393

**Published:** 2025-05-28

**Authors:** Chirine Brinsi, Yassin Khadraoui, Mariem Mhimdi, Slimen Selmi, Houcem Sammari, Saber Jedidi, Houcine Selmi, Hichem Sebai

**Affiliations:** ^1^ Laboratory of Functional Physiology and Valorization of Bio‐Resources (LR23ES08) University of Jendouba, Higher Institute of Biotechnology of Béja Béja Tunisia; ^2^ Laboratory of Sylvo‐Pastoral Resources University of Jendouba, Institution of Agricultural Research and Higher Education (IRESA), Sylvo‐Pastoral Institute of Tabarka Tabarka Tunisia

**Keywords:** *Anethum graveolens* L., inflammation, oxidative stress, rats, ulcerative colitis

## Abstract

Nutritherapy is the art of using nutrients as a means of preventing or correcting dysfunction in the body. We aimed to evaluate the protective effect of dill fruit aqueous extract (DFAE) against acetic acid‐induced ulcerative colitis. Healthy male rats (*N* = 42) were divided into seven groups of six animals each, with groups 1 and 2 being controls, groups 3, 4, and 5 given increasing doses of DFAE (50, 100, and 200 mg/kg, *b.w.*, *p.o*.), and groups 6 and 7 were given sulfasalazine (100 mg/kg, *b.w.*, *p.o.*) and gallic acid (50 mg/kg, *b.w.*, *p.o.*), respectively, during 10 days. In vitro, we found that DFAE's IC_50_ exhibited a significant antioxidant capacity due to its high phenolic content. The in vivo studies showed that the pre‐treatment with DFAE significantly reduced AA‐induced morphological and histopathological alterations of the colonic mucosa. The antioxidant and anti‐inflammatory power of DFAE maintained the redox equilibrium and preserved the intracellular mediators' homeostasis. Our data may suggest that dill fruit is a promising candidate for treating ulcerative colitis by controlling oxidative stress and inflammation.

## Graphical abstract

In this graphical abstract, we demonstrated the protective effects of dill fruit against acute acetic acid‐induced ulcerative colitis due to its antioxidant and anti‐inflammatory properties.

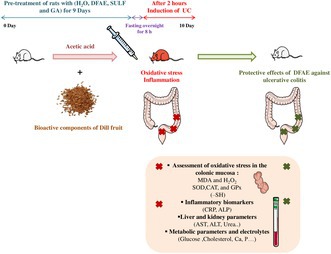

## 1 INTRODUCTION

Inflammatory bowel disease is a pathology corresponding to chronic inflammation of the gastrointestinal system, which develops with episodes of inflammation of varying duration (symptomatic phase), interrupted by phases of remission (asymptomatic phase). These conditions combine two principal diseases: Crohn's and ulcerative colitis (Long, 2024).

Ulcerative colitis is a chronic, relapsing inflammatory disease of the colon characterized by persistent, diffuse inflammation confined to the colonic mucosa and extending into the proximal part of the rectum (Lynch & Hsu, 2024). The main risk factors for UC are genetics, environmental factors, autoimmunity, and intestinal microbiota. Typical symptoms include bloody diarrhea with or without mucus, rectal urgency, tenesmus, and abdominal pain (Gajendran et al., 2019). Current treatment of ulcerative colitis relies mainly on traditional drugs such as aminosalicylates, corticosteroids, and immunosuppressants (Liu et al., 2017). These drugs cannot effectively control the progression of the disease over the long term and are associated with various complications and serious side effects (Xue et al., 2023). Recent studies have shown that a significant increase in interleukin‐1β, IL‐6, tumor necrosis factor‐alpha (TNF‐α), IL‐12, and interferon‐gamma (IFNγ) induced by oxidative stress in IBD may be associated with increased mucosal inflammation (Vebr et al., 2023). Severity has been shown to correlate with nuclear factor kappa B, which plays an essential role in the release of these cytokines and the activation of ulcerative colitis (Amirshahrokhi, 2019). Most IBD patients respond well to conventional treatments. Aminosalicylates are the first choice of treatment for mild‐to‐moderate ulcerative colitis. The problem is that the harmful side effects associated with taking corticosteroids and immunosuppressants, such as sepsis, osteoporosis, cataracts, and mood disorders, cannot be ignored and have a worrying impact on patients' quality of life (Farraj et al., 2022). Currently, almost 40% of UC patients are treated with natural herbal products and functional foods. Flavonoids have achieved significant efficacy in the treatment of ulcerative colitis. Pharmacological mechanisms are linked to the suppression of inflammation, promotion of mucosal healing, maintenance of intestinal immune homeostasis, and control of intestinal microbiota (Xue et al., 2023).

Nutritherapy is a discipline that incorporates the latest discoveries in human physiology and nutrition. In addition to genetics, nutritional approaches also value other factors such as eating habits, eating behavior, physical activity, the microbiome, and the metabolome (Lagoumintzis & Patrinos, 2023). Nutritional therapy aims to prevent or correct disease through nutritional supplements (Alfieri et al., 2023). Bioactive compounds are secondary plant substances in foods that can modulate metabolic processes. In general, these compounds are present in small quantities and are mainly found in plant foods such as fruits, vegetables, and whole grains (Samtiya et al., 2021).

*Anethum graveolens* L. (commonly known as Dill) belongs to the Apiaceae family and is a plant used as a medicine and spice (Saleh‐e‐In et al., 2017). The best source of antioxidants, including vitamin C, polyphenols, and carotenoids, is dill (Mobasseri et al., 2014). It has anticancer, antibacterial, antigastric, anti‐inflammatory, and antioxidant properties (Oshaghi et al., 2016). Because of the plant's wide‐ranging therapeutic effects, it can be used as an analgesic to relieve headaches and to reduce inflammation of the stomach, intestine, and bladder. Some studies suggested that components of this plant inhibit the release of inflammatory mediators and reduce the cyclooxygenase 1 and 2 pathways (Payahoo et al., 2014). In a recent study, dill fruit used for gastrointestinal disorders had beneficial effects against diarrhea, one of the repercussions of ulcerative colitis (Brinsi et al., 2024). Each part of the fruit called the schizocarp forms a seed that fuses to the fruit. These seeds are traditional medicines to treat various stomach, liver, kidney, and brain diseases (Naseri et al., 2012).

**FIGURE 1:**
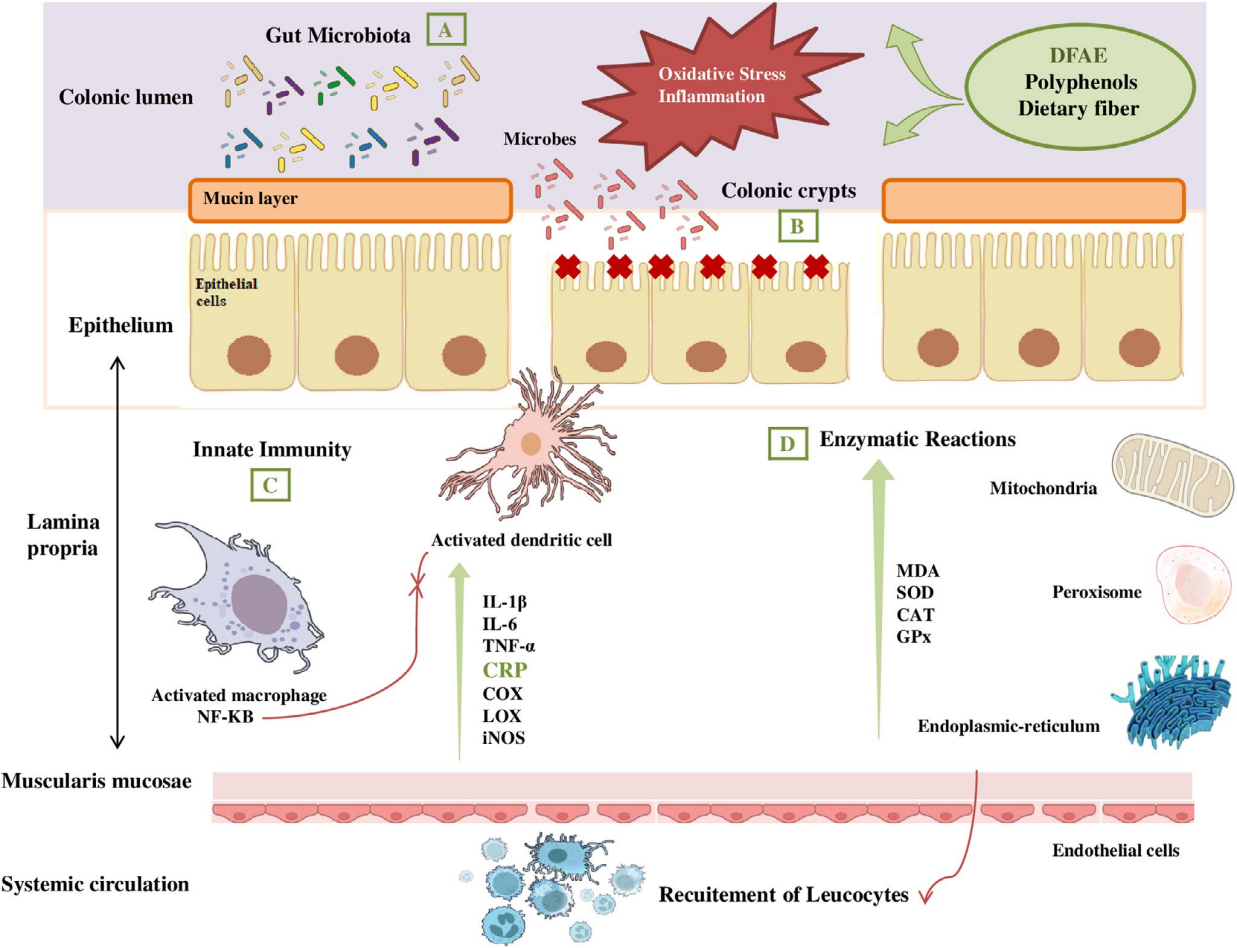
The protective effect of Dill fruit aqueous extract (DFAE) on the pathophysiology of ulcerative colitis. (a) Regulation of gut microbiota dysbiosis; (b) restoration of the intestinal barrier; (c) positive regulation of immune response; (d) improving the balance of antioxidant defense systems.

This investigation aimed to demonstrate the potential benefits of dill fruit extract (DFAE) as a natural, safe, and effective alternative treatment for ulcerative colitis (Figure 1).

## 2 MATERIALS AND METHODS

### 2.1 Reagents and chemicals

Acetic acid (PubChem CID:176), bovine catalase (PubChem CID: 135337101), alcian blue (PubChem CID:76418923), epinephrine (PubChem CID: 5816), GSH (PubChem CID: 124886), butylated hydroxytoluene (PubChem CID: 31404), gallic acid (PubChem CID: 370), 2‐Thio‐barbituric acid (PubChem CID: 7559), trichloroacetic acid (PubChem CID: 23681045), hydrochloric acid (PubChem CID: 313), magnesium chloride (PubChem CID: 5360315), methanol (PubChem CID: 18177619), sodium acetate (PubChem CID: 517045), sodium hydroxide (PubChem CID:14798), cetrimonium bromide (PubChem CID:5974), and sulfuric acid (PubChem CID:118) were from Sigma Chemical Co.

Hydrogen peroxide (H_2_O_2_) and sulfasalazine were purchased from Tunisia's central pharmacy. All other chemicals used were of analytical grade.

### 2.2 Phytochemical screening of dill fruit

#### 2.2.1 Determination of parietal constituent content

The determination of parietal constituents such as neutral detergent fiber (NDF), acid detergent fiber (ADF), and acid detergent lignin (ADL) of samples was carried out according to the method of (Van Soest et al., 1991) using a semiautomatic FIBRESTEST RAYPA device.

##### Neutral detergent insoluble fibers (NDF)

Extraction was performed by boiling a 1 g powder sample in 100 mL NDS solution for 1 h. Grease and ash insoluble in mild detergents were removed from the residue by rinsing with acetone and hot distilled water.

##### Acid‐insoluble fibers (ADF)

This ratio was determined by hydrolysis of the sample in the presence of a sulfuric acid solution and a surfactant (CTAB = cetyltrimonium bromide). Hydrolysis of the sample (1 g of powder in 100 mL of solution in a sintered glass crucible) takes place by boiling for 1 h. Grease (rinsed with acetone) and insoluble ash (calcined for 4 h at 550°C in a muffle furnace) are removed from the residue. The ADF fraction corresponds to true cellulose and lignin.

##### Sulfuric lignin (ADL)

The crucible containing the ADF residue was collected and treated with H_2_SO_4_ (72%). The mixture was incubated for 3 h at room temperature. The crucible was then filtered under vacuum through a filter lamp, and the residue was washed several times with hot distilled water. The crucible was then dried at 105°C for 12 h, the dry weight measured, and the residue calcined at 550°C for 4 h.

The cell content was solubilized by the action of sodium lauryl sulfate detergent and pectic substances by a chelating agent in a buffered medium, resulting in the formation of the cell wall (NDF). Theoretically, the fraction consists of cellulose, hemicelluloses, and lignin. Sulfuric acid was used to determine the ADL content by treating the ADF fraction, where the true raw cellulose content (TRC) represented the difference between ADF and ADL contents. The amount of hemicellulose (HC) was calculated based on the difference between NDF and ADF.

#### 2.2.2 Preparing of dill fruit aqueous extract

Dill fruits were harvested from early June to September in the Beja region and supplied by farmers. The specimen (No. AG 352) has been placed in the barium of the Higher Institute of Biotechnology of Beja (University of Jendouba, Tunisia). After drying in ambient air in the dark, the dill fruits were ground. The maceration was made with distilled water (1/20; w/v) at 45°C for 24 h under magnetic agitation and the homogenate was filtered with a 0.45 μm Whatman #1 filter paper (Bärenstein, Germany), and the extraction yield (%) was measured. The dry remains were weighed and absorbed into 3 mL of distilled water, subsequently used for the phytochemical analysis and the in vivo experiments.

#### 2.2.3 Secondary metabolites determination of DFAE

Total polyphenols were measured using the Folin–Ciocalteu assay according to the Singleton method (Vl, 1999). Briefly, 500 μL of the extract was added to 10 mL of water and 0.5 mL of Folin–Ciocalteu reagent. After 5 min, 8 mL of 7.5% sodium carbonate solution was added. The reaction mixture was kept in the dark for 2 h, and absorbance was measured at 760 nm. Gallic acid (GA) was used as the standard, and results were expressed as mg GA equivalent per gram of dry matter (mg GAE/g DM).

Total flavonoid content was determined according to the aluminum chloride (AlCl_3_) colorimetric (Yi et al., 2008). Briefly, 1 mL of the sample was mixed with 1 mL of 2% AlCl_3_ solution. After 15 min of incubation at room temperature, the absorbance of the reaction mixture was read at 430 nm. Quercetin (Q) was used as the standard, and results were expressed as mg Q equivalent per gram of dry matter (mg QE/g DM).

The interaction of flavonoids with the free radical 1,1‐diphenyl‐2‐picrylhydrazyl (DPPH) has been described in several studies. The reactivity of flavonoids with a stable free radical is given by the DPPH assay (Cotelle et al., 1996). DPPH has a broad absorption band in ethanol at 517 nm due to its odd electron.

Flavonol quantification was assessed according to the method of (Geng & Xiang, 2007). Briefly, 1 mL AlCl_3_ (20%) was added to 1 mL extract followed by 3 mL sodium acetate (50 mg/mL). After 2 h and 30 min incubation, absorbance was read at 440 nm. Rutin was used as the standard and results were expressed as mg rutin equivalents per gram of dry matter (mg RE/g DM).

Total tannins were determined using the Folin–Ciocalteu reagent (Kujala et al., 2000). 0.5 mL FolinCiocalteu (50%) was added to 0.5 mL extract, followed by 1 mL Na_2_CO_3_ (20%). The absorbance of the supernatant was measured at 730 nm using a visible ultraviolet (UV) spectrophotometer (DU 640B, Beckman Coulter).

Condensed tannin was determined according to the modified vanillin assay (Price et al., 1978). Briefly, 250 μL of sample (10%, w/v) was added to 750 μL of water, 1.5 mL of 4% vanillin solution, and 750 μL of HCl (12 N). The reaction mixture was kept in the dark for 20 min, after which absorbance was measured at 500 nm. Catechin was used as the standard, and results were expressed as mg catechin equivalents per gram of dry matter (mg CE/g DM).

Total anthocyanins were estimated by absorbance differentiation and using two buffers (KCl and CH_3_COONa). Briefly, 400 μL of extract was mixed with 3.6 mL of KCl, followed by 400 μL of CH_3_COONa. After 30 min of incubation in the dark, the absorbance of the reaction mixture was read at 510 nm (Lee et al., 2005). Cyanidin3glycosyl (CG) was used as the standard, and results were expressed as mg CG per gram dry matter (mg CGE/g DM).

### 2.3 Free radical scavenging activity on DPPH ^•^

The antioxidant activity of dill extract was determined by the DPPH^•^ radical scavenging assay (Ammar et al., 2009). 1 mL of the aqueous extract at different concentrations (10, 25, 50, 100, 150, 200, and 250 μg/mL) was added to 1 mL of the methanolic solution of DPPH 0.06 mM (2.4 mg/100 mL). At the same time, a negative control was prepared by mixing 1 mL of distilled water with 1 mL of the DPPH solution and the BHT solution used as a reference molecule. As a result, the violet color of DPPH turns yellow after reduction by the extract, and this discoloration can be measured spectrophotometrically. The mixture was kept at room temperature in the dark for 30 min, and the optical density was measured at 517 nm.

Inhibition percentages were estimated according to the following equation:

$$I\%=\left(\left(\mathrm{Abs}\;\text{control}-\mathrm{Abs}\;\text{test}\right)/\mathrm{Abs}\;\text{control}\right)\times 100$$

### 2.4 HPLC‐DAD analysis of dill fruit aqueous extract

Identification of phenolic compounds in the aqueous extract of dill fruits was carried out using an HPLC instrument (Agilent 1260, Agilent Technologies, Germany) equipped with a diode array detector (DAD), vacuum degasser, autosampler, and binary pump with a maximum pressure of 400 bar. Separations were performed at 25°C on a Zorbax Eclipse XDB C18 reversed‐phase analytical column (3.5 μm, 4.6 × 100 mm). Methanol (solvent A) and 0.1% formic acid (solvent B) made up the mobile phase. The injected sample volume was 20 μL and DAD was monitored at 254 nm to detect phenolic compounds in the extract, identified according to their retention time (RT).

### 2.5 Animals and treatment

The study involved 42 Male Wistar rats weighing (150–250 g; *n* = 6). Animals were housed six per group per cage according to the following measurements: length × width × height = 37 × 21 × 15 cm and from the Society of Pharmaceutical Industries of Tunisia (SIPHAT, Ben‐Arours, TN). Animals were acclimatized for 2 weeks with a standard pellet diet (Badr‐Utique‐TN), water ad libitum, and maintained in the animal house at a controlled temperature (22 ± 2°C; 12 h dark/light cycle). The daily photoperiod is as follows: lights on from 8 h to 20 h and off from 20 h to 8 h. All maintenance and sacrifice procedures were used following the local ethics committee of Tunis University regarding the use and care of animals and by the NIH recommendation. The protocol was approved by the “Comité d'Ethique Bio‐medicale (CEBM)” (JORT472001) of the “Institut Pasteur de Tunis.”

The rats were divided into seven groups of six animals each: Group 1 served as control and received distilled water (5 mL/kg, *b.w*., *p.o*.). Group 2 served as AA group and received distilled water (5 mL/kg, *b.w*., *p.o*.). Groups 3, 4, and 5 were pretreated with different doses of DFAE (50, 100, and 200 mg/kg, *b.w*., *p.o*.). Preliminary experiments indicated that doses of (50, 100, and 200 mg/kg) of DFAE were the lowest that gave a significant protective effect. Groups 6 and 7 received sulfasalazine and gallic acid at (100 and 50 mg/kg, *b.w*., *p.o*.), respectively. SULF and GA powders were dissolved in distilled water.

Pretreatment of the animals with (H_2_O, DFAE, SULF, and GA) lasted 9 days. All these treatments were administered by gavage.

### 2.6 Induction of ulcerative colitis

All animals were fasted overnight for 8 h. Then after 2 h (10 days), G2, 3, 4, 5, 6, and 7 all received rectal administration of AA, except G1, which was treated with (10 mL/kg, *b.w*., *p.o*.) physiological solution (NaCl, 0.9%, *p.o*.). Ulcerative colitis was induced by injection of acetic acid (3%, v/v, 5 mL/kg, *b.w*.) for 30 s through a polyethylene tube inserted into the rectum at a distance of 8 cm, leading to the infusion of acetic acid in the colon. After 24 h, rats were anesthetized by intraperitoneal administration of sodium pentobarbital (40 mg/kg, *b.w*.) and sacrificed by decapitation (Hajji et al., 2018). The colon was immediately excised and examined macroscopically, and colonic mucosa samples were incubated with phosphate‐buffered saline (PBS), homogenized, and centrifuged at 10000*g* for 15 min. The supernatant was stored at −80°C for biochemical parameters and assessment of oxidative status. The remaining colon was preserved in 10% formalin for histopathological examination. Blood was also collected in heparinized tubes. After centrifugation at 3000*g* for 15 min, plasma was stored at −20°C for biochemical measurements.

### 2.7 Assessment severity of colitis

For each animal, the distal part of the colon was removed, cut longitudinally, and then washed with saline solution to remove fecal debris. Lesions in the colonic mucosa were examined macroscopically, and photographs of hemorrhagic erosions were taken with a Canon EOS1100 D digital camera (ISO 6400). The ulcer index was determined as the sum of the lengths of all gastric lesions in (mm^2^).

### 2.8 Histological analysis

Immediately after euthanasia, distal colon segments were fixed in 10% neutral buffered formalin, embedded in paraffin, and used for histopathological examination. Sections 4 μm thick were cut, deparaffinized, hydrated, and stained with hematoxylin and eosin (H&E). In all treatments, colon sections were examined blind.

### 2.9 Assessment of oxidative activity

#### 2.9.1 Measurement of lipid peroxidation and determination of H_2_O_2_

Lipid peroxidation in colonic mucosa was determined by measuring MDA using double heating (Draper & Hadley, 1990). Briefly, gastric and intestinal mucosal supernatants were mixed with trichloroacetic acid‐hydroxytoluenebutyric acid (TCA‐BHT) (1% and 20%, w/v, respectively). Centrifuge the mixture at 1000*g* for 5 min at 4°C. The supernatant was added to the buffer (HCl 0.5 N, TBA 120 mM, buffered with Tris 26 mM). The mixture was heated to 80°C for 10 min and then cooled. MDA levels were estimated based on the molar extinction coefficient of MDA‐TBA (equal to 1.56 × 105 M^−1^ cm^−1^) after reading at 532 nm in a spectrophotometer.

The colonic mucosa H_2_O_2_ level was carried out according to (Dingeon et al., 1975). In the presence of phenol and peroxidase, H_2_O_2_ reacts with 4‐aminoantipyrine and P‐hydroxybenzoic acid to form a pale pink complex whose absorbance was measured at 505 nm. This was calculated using a calibration curve drawn from a series of standard hydrogen peroxide solutions.

#### 2.9.2 Antioxidant enzyme activities

Superoxide dismutase (SOD) activity in colonic mucosa was determined using modified epinephrine assays (Kakkar et al., 1984). Superoxide anion induces the autoxidation of epinephrine to adenochrome in a basic medium. In this reaction, SOD competed to form H_2_O_2_ from the superoxide anion and prevented the formation of this colored complex. Catalase reacted to produce water from H_2_O_2_ and prevented the formation of superoxide anions. One unit of SOD was defined as the amount of extract that inhibits the rate of adenochrome formation by 50%. Briefly, the enzyme extract was mixed with 10 μL bovine catalase (0.4 U/mL), 20 μL epinephrine (5 mg/mL), and 62.5 mM carbonate/bicarbonate buffer (pH 10.2). Changes in absorbance were evaluated at 480 nm.

CAT activity was assessed by measuring initial H_2_O_2_ disappearance at 240 nm (Aebi, 1984). Briefly, 50 mM phosphate buffer (pH 7) was added to the extract, and the decrease in absorbance was recorded after the addition of 33 mM H_2_O_2_. Optical density was assessed every 30 s for 2 min at 240 nm. Catalase levels were calculated using the H_2_O_2_ molar extinction coefficient of 40 mM^−1^ cm^−1^, and concentrations were expressed as μmol H_2_O_2_ per milligram of protein.

Glutathione peroxidase (GPx) activity was quantified according to the procedure of Flohé and Günzler (1984). H_2_O_2_ reacts with reduced glutathione (GSH) under the action of GPx to form GSSG. For each test portion, 0.2 mL enzyme extract, 0.2 mL (GSH, 0.1 Mm), 0.2 mL phosphate buffer (0.1 M, pH 7.4), and 0.4 mL mixture (H_2_O_2_, 5 mM) were added continuously. The obtained mixture was incubated at 37°C for 1 min and added to 0.5 mL TCA (5%) to block the reaction. The mixture was centrifuged (5 min; 1500*g*), 0.2 mL supernatant was collected and mixed with 0.5 mL of phosphate buffer (0.1 M, pH 7.4) and 0.5 mL of DTNB (10 Mm). Absorbance was expressed at 412 nm and GPx concentration was expressed as mM GSH/min/mg protein.

#### 2.9.3 Nonenzymatic antioxidants

Total concentrations of thiol groups (‐SH) in colonic mucosa were determined using Ellman's method (Ellman, 1959). Accordingly, for each test, the enzyme extract was mixed with 0.8 mL phosphate buffer (0.25 M, pH 8.2) and 0.1 mL of EDTA (20 mM). The mixture was vortexed and its absorbance was measured at 412 nm (A1). Next, 100 μL of DTNB (10 mM) was added and incubated for 15 min at 37°C, and a second absorbance was recorded on a blank (B) containing 1050 μL of buffer and 20 μL of DTNB (A2). The difference between (A2), (A1), and (B) is used to determine the concentration of (‐SH), and the result was expressed in nmol thiol groups/mg protein.

### 2.10 Determination of inflammatory biomarkers

These parameters were measured using commercially available diagnostic kits (Biosystems S.A. Barcelona, Spain) (ISO 9001). C‐reactive protein content (COD 31029) and alkaline phosphatase activity (COD 11592).

### 2.11 Determination of renal and hepatic parameters

Uree (COD 11517), ALT (COD 11533), AST (COD 11531), and Creatinine (COD 11502) were measured using commercially available diagnostic kits (Biosystems S.A. Barcelona, Spain) (ISO 9001).

### 2.12 Determination of metabolic parameters and electrolytes

Glucose (COD 11504), Cholesterol (COD 11506), Triglycerides (COD 11529), Calcium (COD 11570), Magnesium (COD 11797), and Phosphorus (COD 11508) were measured using commercially available diagnostic kits (Biosystems S.A. Barcelona, Spain) (ISO 9001).

### 2.13 Statistical analysis

Data were analyzed by a post hoc Tukey analysis of variance Anova One Way using IBM SPSS Statistics for Windows, version 25 (IBM Corp., Armonk, N.Y., USA) and were expressed as mean ± standard deviation of the mean (SD). All statistical tests were two‐tailed, and a *p* value of 0.05 or less was considered significant.

## 3 RESULTS

### 3.1 Parietal constituents and secondary metabolite

By analyzing the parietal constituents, we found that the dill fruit contains high levels of insoluble fiber (Table 1) such as neutral detergent fiber (60.77 ± 0.60%), hemicelluloses (39.11 ± 2.5%), and crude lignin (17.19 ± 1.84%).

**TABLE 1:** Parietal composition (% DM) of dill fruit.

Parameters	Contents
% Neutral detergent fiber	60.77 ± 0.34
% Acid detergent fiber	21.66 ± 1.82
% Acid detergent lignin	18.94 ± 1.67
% Hemicellulose	39.11 ± 1.47
% True raw cellulose	2.72 ± 0.15
% Crude lignin	17.19 ± 1.06

*Note*: Data are represented as mean ± SD (*n* = 3).

The quantification of secondary metabolites showed that dill fruit aqueous extract (DFAE) had high levels of polyphenols (380.43 ± 1.30 mg GAE/g DM), flavonoids (39.27 ± 0.41 mg QE/g DM), and total tannins (233.77 ± 6.47 mg TAE/g DM) (Table 2).

**TABLE 2:** Phytochemical composition and IC_50_ value of the DPPH• radical of dill fruit aqueous extract (DFAE) with reference molecules.

Composition	DFAE contents
Total phenolics (mg GAE/g DM)	380.43 ± 0.75
Flavonoids (mg QE/g DM)	39.27 ± 0.24
Flavonols (mg RE/g DM)	18.44 ± 0.52
Total tannins (mg TAE/g DM)	233.77 ± 3.73
Condensed tannins (mg CE/g DM)	0.006 ± 0
Total anthocyanins (mg CGE/g DM)	0.24 ± 0.006
DPPH IC_50_ (μg/mL)	41.71 ± 1.29
BHT IC_50_ (μg/mL)	41.04 ± 1.26
Ascorbic acid IC_50_ (μg/mL)	26.84 ± 0.1

*Note*: Data are expressed as mean ± SD (*n* = 3 of independent DFAE preparations).

Abbreviations: CE, catechin equivalent; CGE, cyanidine glucosyl‐3 equivalent; DM, dry matter; GAE, gallic acid equivalent; IC_50_, inhibitory concentration; QE, quercetin equivalent; RE, rutin equivalent; TAE, tannic acid equivalent.

### 3.2 Bioactive compounds profile and antioxidant capacity of DFAE

Dill fruit exhibited a significant dose‐dependent antioxidant capacity (Table 2). The IC_50 of_ DFAE estimated at (IC_50_ = 41.71 ± 2.23 μg/mL) is close to that of Ascorbic Acid (IC_50_ = 26.84 ± 0.1 μg/mL) and BHT (IC_50_ = 41.04 ± 1.26 μg/mL), which were used as a reference antioxidant molecules.

**FIGURE 2:**
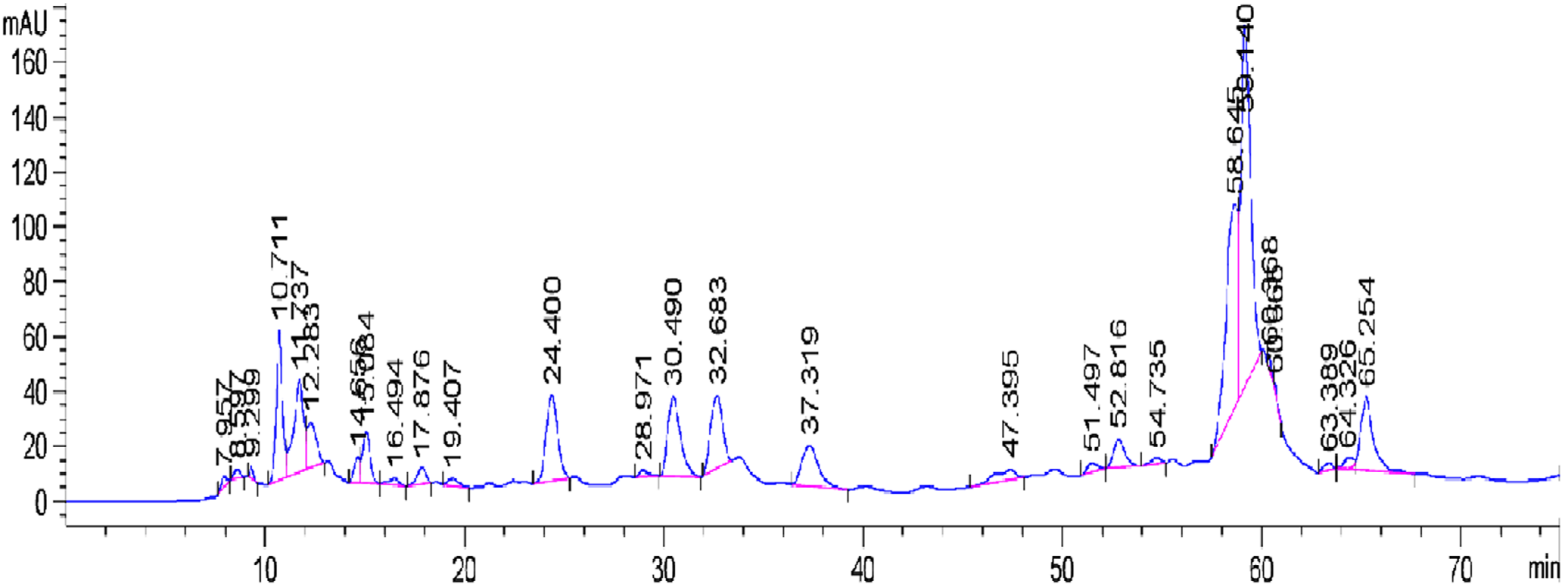
Chromatogram of aqueous extract of *Anethum graveolens* fruit (DFAE) (peak distribution shown in Table 2).

HPLC‐DAD analysis of DFAE identified 27 phenolic compounds. The results showed 14 main phenolic compounds, 7 phenolic acids, and 7 flavonoids. The main compounds are flavonoids, notably quercetin and resveratrol (24.19% and 16.73%), respectively (Figure 2 and Table 3).

**TABLE 3:** Main phenolic compounds identified in the aqueous extract of dill fruit.

Peak	Identified compound	Molecular formula	Molecular weight (g/mol)	PubChem CID	RT (min)	Composition (%)
1	Gallic acid	C_7_H_6_O_5_	170.12	370	7.957	0.3748
2	Chlorogenic acid	C_16_H_18_O_9_	354.31	1794427	8.597	0.3770
3	Protocatechic acid	C_7_H_6_O_4_	154.12	72	9.299	0.2324
4	Caffeic acid	C_9_H_8_O_4_	180.16	689043	10.711	6.2362
5	Epicatechin	C_15_H_14_O_6_	290.27	72276	11.737	6.1958
8	Coumaric acid	C_9_H_8_O_3_	164.16	637542	15.084	2.6195
12	Ferulic acid	C_10_H_10_O_4_	194.18	445858	24.400	6.7462
14	Rutin	C_27_H_30_O_16_	610.5	5280805	30.490	6.8047
15	Rosmarinic acid	C_18_H_16_O_8_	360.3	5281792	32.683	5.8912
16	Kaempferol‐3‐Oglucoside	C_21_H_19_O_11_	447.4	25203515	37.319	4.6488
17	Isorhamnetin‐3‐O‐glucoside	C_22_H_22_O_12_	478.4	5318645	47.395	1.5791
21	Resveratrol	C_14_H_12_O_3_	228.24	445154	58.645	16.7312
22	Quercetin	C_15_H_10_O_7_	302.23	5280343	59.140	24.1991
27	Kaempherol	C_15_H_10_O_6_	286.24	5280863	65.254	6.4622

### 3.3 Qualitative and quantitative assessment of colon lesions

**FIGURE 3:**
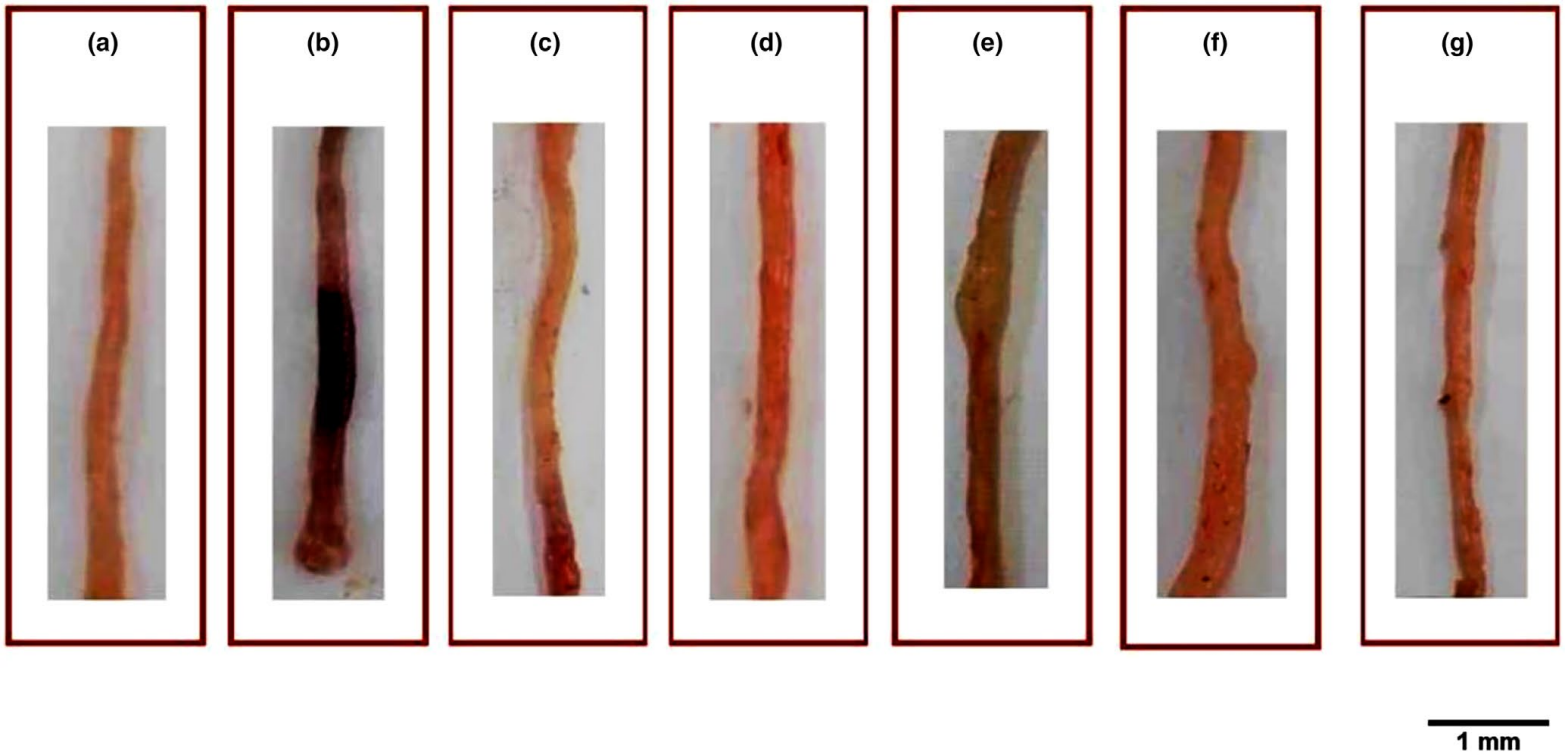
Effects of dill fruit aqueous extract (DFAE), sulfasalazine (SULF), and gallic acid (GA) against the increase in colonic lesions induced by acetic acid in rats. Animals were pretreated with various doses of DFAE (50, 100, and 200 mg/kg, *b.w*., *p.o*.), SULF (100 mg/kg, *b.w*., *p.o*.), or GA (50 mg/kg, *b.w*., *p.o*.) or distilled water. Rats were challenged with a single anal administration of AA (3%, v/v, 5 mL/kg, *b.w*.) or NaCl (0.9%, 5 mL/kg, *b.w*.) for 24 h. (a): H_2_O + NaCl, (b): H_2_O + AA, (c): DFAE (50 mg/kg, *b.w*., *p. o*.) + AA, (d): DFAE (100 mg/kg, *b.w*., *p.o*.) + AA, (e): DFAE (200 mg/kg, *b.w*., *p.o*.) + AA, (f): SULF (100 mg/kg, *b.w*., *p.o*.) + AA, and (g): GA (50 mg/kg, *b.w*., *p.o*.) + AA.

The macroscopic signs of ulcerative colitis include alteration of the intestinal barrier, hemorrhage, and edema of the colonic mucosa as opposed to the healthy control group. The pretreatment with varied doses of DFAE (50, 100, and 200 mg/kg, *b.w*., *p.o*.) significantly (*p* < 0.05) and dose‐dependently reduced these disorders compared with the colitis of the control group as demonstrated in Figure 3. This cytoprotective effect can be explained in the group pre‐treated with the highest dose of DFAE, which is comparable to that of sulfasalazine (standard drug) and gallic acid (reference antioxidant molecule).

Our results demonstrated that anal administration of AA‐induced increase in wet colon weight and ulcerated surface scores compared with the pre‐treated groups DFAE, SULF, and GA as shown in Table 4.

**TABLE 4:** Protective effects of dill fruit aqueous extract (DFAE), sulfasalazine (SULF), and gallic acid (GA) on colonic mucosal structure and colon weight to length ratio following AA‐induced ulcerative colitis in rats. Animals were pretreated with various doses of DFAE (50, 100, and 200 mg/kg, *b.w.*, *p.o*.), SULF (100 mg/kg, *b.w.*, *p.o*.), or GA (50 mg/kg, *b.w.*, *p.o*.) and distilled water. Rats were challenged with a single anal administration of AA (3%, v/v, 5 mL/kg, *b.w*.) or NaCl (0.9%, 5 mL/kg, *b.w*.) for 24 h.

Treatment	Macroscopic lesion score (mm^2^)	% protection	Wet colon weight/length (mg/cm)	% protection
Control	–	–	75.93 ± 3.15	–
AA	303 ± 0.14*	–	111.14 ± 5.17*	–
AA + DFAE‐50	199 ± 0.04^#^	34.37	87.74 ± 3.38^#^	21.05
AA + DFAE‐100	123 ± 0.02^#^	59.36	84.97 ± 4.29^#^	23.54
AA + DFAE‐200	117 ± 0.09^#^	61.23	79.91 ± 2.99^#^	28.09
AA + SULF	121 ± 0.09^#^	59.85	81.98 ± 2.45^#^	26.23
AA + GA	162 ± 0.05^#^	46.51	85.93 ± 3.99^#^	22.68

*Note*: Data are expressed as means ± SD (*n* = 6).

* *p* < 0.05 compared with control group.

^#^ *p* < 0.05 compared with AA group.

### 3.4 Histopathological study

**FIGURE 4:**
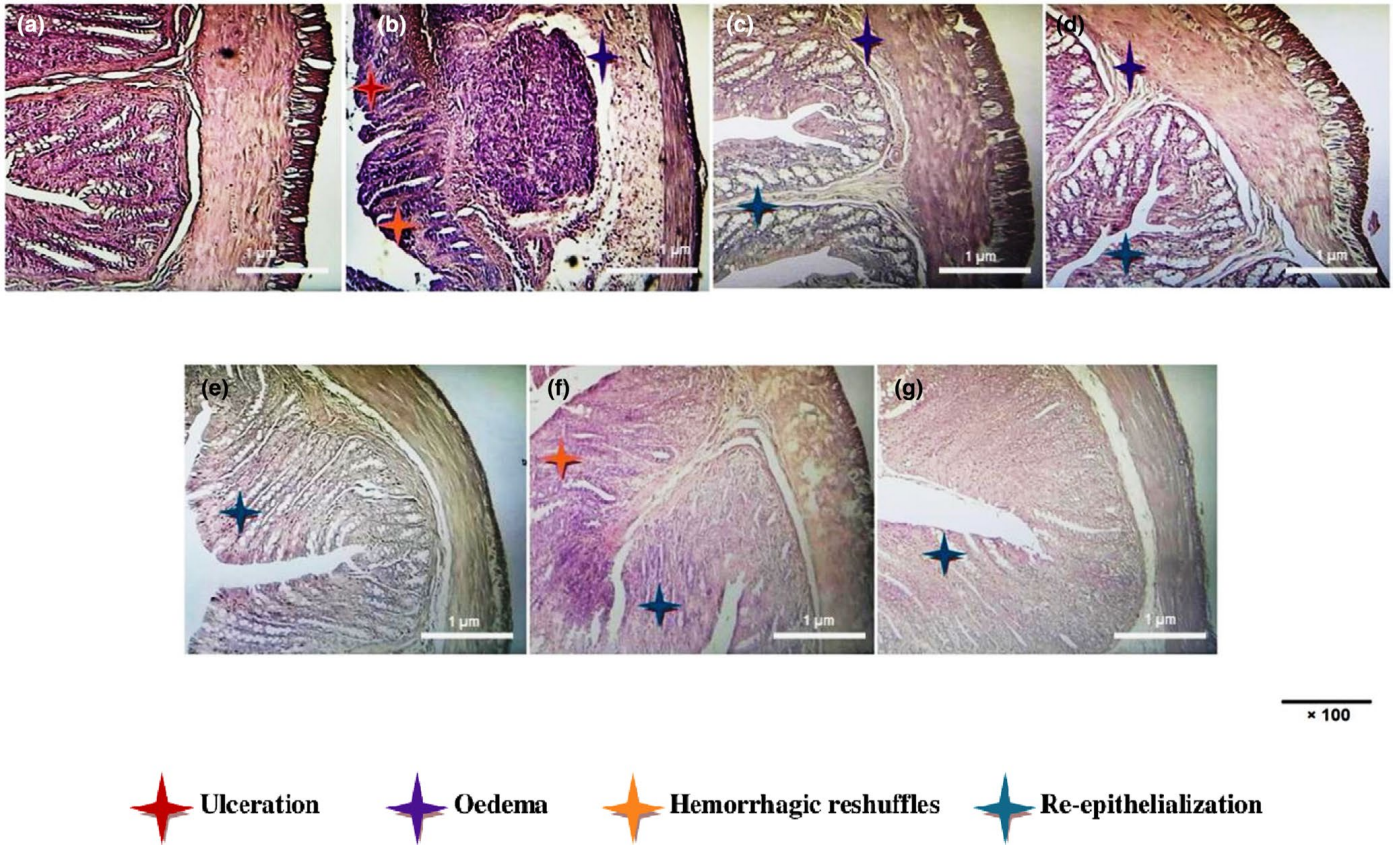
Effects of dill fruit aqueous extract (DFAE), sulfasalazine (SULF), and gallic acid (GA) on histological changes in colonic mucosa following intrarectal intoxication with acetic acid in rats. Animals were pretreated with various doses of DFAE (50, 100, and 200 mg/kg, *b.w*., *p.o*.), SULF (100 mg/kg, *b.w*., *p.o*.), or GA (50 mg/kg, *b.w*., *p.o*.) or distilled water. Rats were challenged with a single anal administration of AA (3%, v/v, 5 mL/kg, *b.w*.) or NaCl (0.9%, 5 mL/kg, *b.w*.) for 24 h. (a): H_2_O + NaCl, (b): H_2_O + AA, (c): DFAE (50 mg/kg, *b.w*., *p.o*.) + AA, (d): DFAE (100 mg/kg, *b.w*., *p.o*.) + AA, (e): DFAE (200 mg/kg, *b.w*., *p.o*.) + AA, (f): SULF (100 mg/kg, *b.w*., *p.o*.) + AA, and (g): GA (50 mg/kg, *b.w*., *p.o*.) + AA. H&E, ×100, scale bar 1 μm.

As demonstrated in the photograph of colonic sections, intra‐rectal administration of acetic acid (3%, v/v, 5 mL/kg, *b.w*.) shows several complex histological lesions identified in the mucosa such as congestion of vascular and epithelial cells of the mucosa and submucosa, alteration of the surface coating, edema, necrosis, and diffuse infiltration of inflammatory cells in the ulcerative colitis group, showed in Figure 4. The structure and number of crypts were also affected by their reduction and the erosion of their upper part. Our results also showed damage at the cellular level expressed by acute necrosis, apoptosis, and mucosal ulcerations affecting colonic tissues to varying degrees.

However, pretreatment with DFAE considerably exerted remarkable protection against AA‐induced deterioration of colonic mucosal structure such as regression of hemorrhagic foci, lesions, and congestion, and preservation of crypt shape and length in a dose‐dependent manner. In addition, pre‐treatment with SULF (100 mg/kg, *b.w*., *p.o*.) and GA (50 mg/kg, *b.w*., *p.o*.) also significantly protected the colonic mucosa against acetic acid‐induced colonic cell damage. The high phenolic compound content of DFAE fruit has been shown to prevent ulcerative colitis while helping the colonic mucosa maintain its structure.

### 3.5 Effects of DFAE on acetic acid‐induced lipid peroxidation and H_2_O_2_ accumulation

**FIGURE 5:**
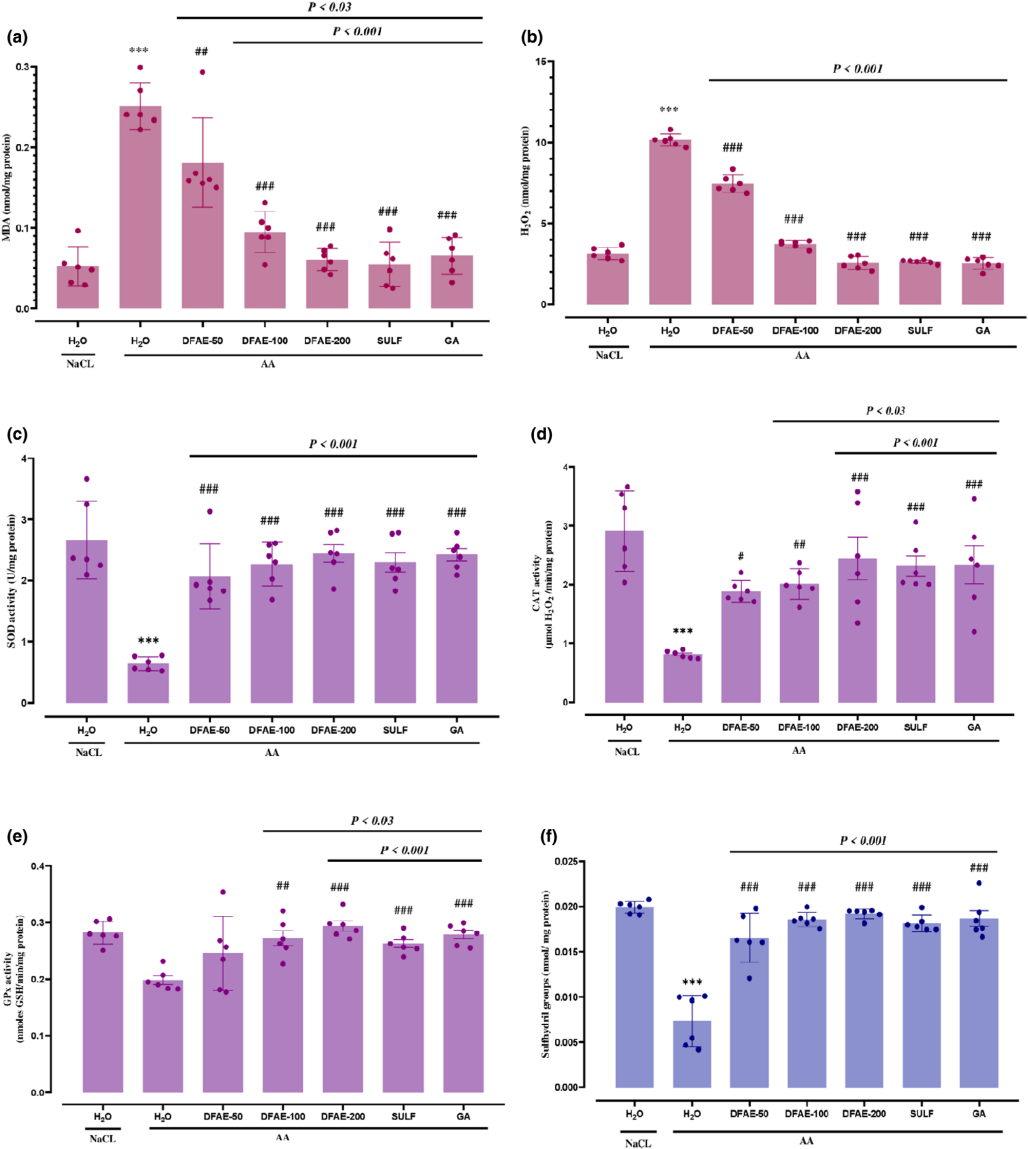
Protective effects of dill fruit aqueous extract (DFAE), sulfasalazine (SULF), and gallic acid (GA) on AA‐induced ulcerative colitis in rats on MDA (a) and hydrogen peroxide (b), on antioxidant enzyme activities: SOD (c), CAT (d), and GPx (e) and sulfhydryl‐SH (f) levels on colonic mucosal. Animals were pretreated with various doses of DFAE (50, 100, and 200 mg/kg, *b.w*., *p.o*.), SULF (100 mg/kg, *b.w*., *p.o*.), or GA (50 mg/kg, *b.w., p.o*.) or distilled water. Rats were challenged with a single anal administration of AA (3%, v/v, 5 mL/kg, *b.w*.) or NaCl (0.9%, 5 mL/kg, *b.w*.) for 24 h. Data are expressed as means ± SD (*n* = 6) in each group. ***p < 0.001 compared to the control group; ^#^ *p* < 0.05, ^##^ *p* < 0.03, and ^*###*^ *p* < 0.001 compared to the acetic acid group. The lines present a significant difference between the two groups shown.

The implication of oxidative stress in the antiulcerogenic effect of DFAE was determined by the levels of MDA and hydrogen peroxide in colonic mucosa. Compared to the control group, MDA and H_2_O_2_ levels were increased significantly (*p* < 0.05). Pre‐treatment with DFAE, SULF, and GA showed significant protection of the colon against lipid peroxidation, as well as a decrease in H_2_O_2_ content compared to the AA group in a dose‐dependent manner (Figure 5a,b).

### 3.6 Effects of DFAE on AA‐induced depletion of antioxidant enzyme activity

Antioxidant enzyme activities (SOD, CAT, and GPx) in the acetic acid group (3%, v/v, 5 mL/kg, *b.w*.) were significantly decreased in the colonic mucosa compared with the control and illustrated in Figure 5c–e. However, pre‐treatment with DFAE, SULF, and GA abolished the depletion of enzyme activities in a dose‐dependent manner (*p* < 0.05).

### 3.7 Effect of DFAE, GA, and SULF on a nonenzymatic antioxidant (‐SH)

The determination of nonenzymatic antioxidant contents demonstrated that AA induced a significant (*p* < 0.05) depletion of the thiol group in the colon compared with control animals, presented in Figure 5f. Furthermore, we registered that the high dose of DFAE exerts a more significant effect than sulfasalazine at (100 mg/kg, *b.w*., *p.o*.) and gallic acid (50 mg/kg, *b.w*., *p.o*.), used as reference molecules.

### 3.8 Effect of DFAE on inflammatory parameters

**FIGURE 6:**
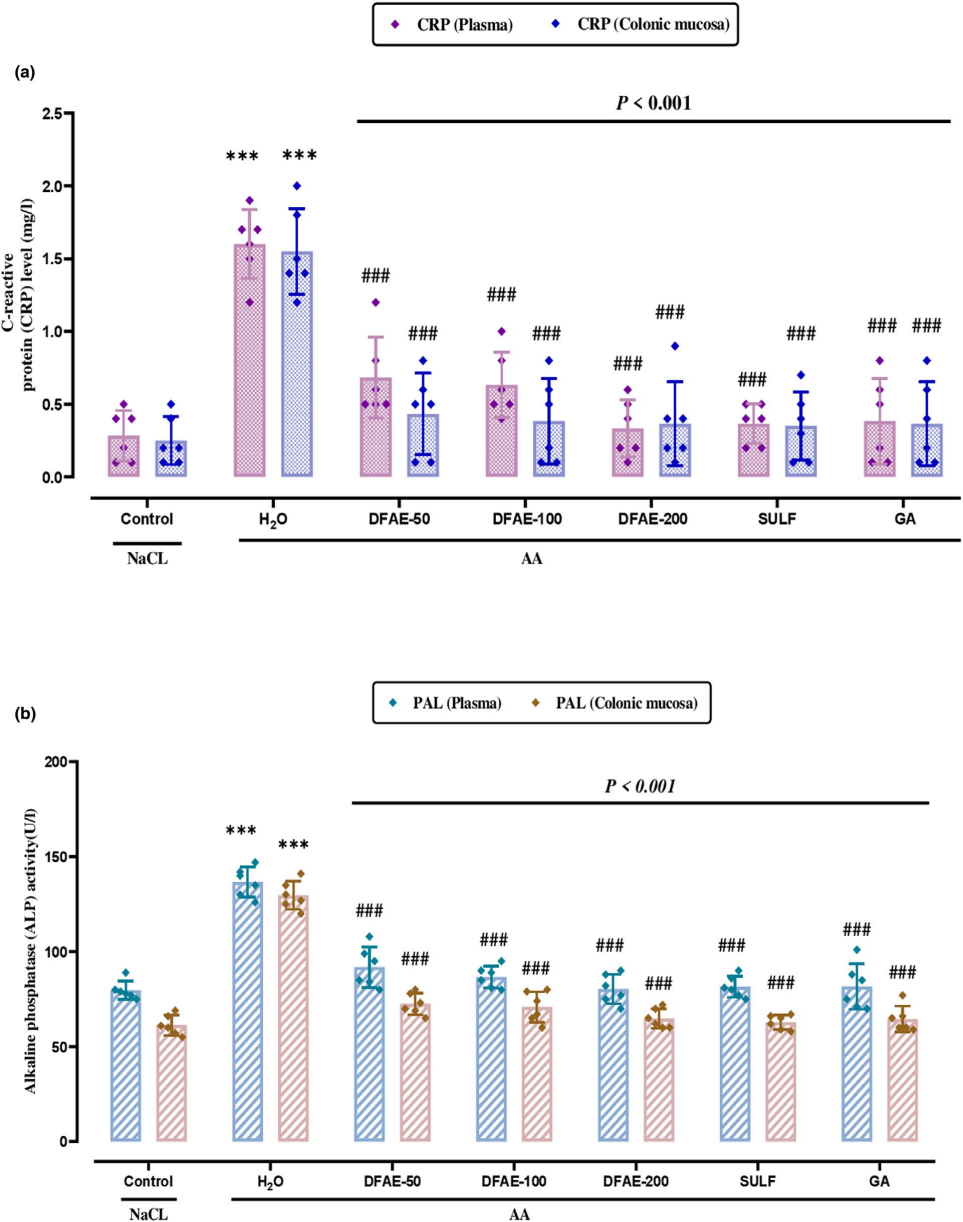
Protective effects of dill fruit aqueous extract (DFAE), sulfasalazine (SULF), and gallic acid (GA) on inflammatory biomarkers in CRP (a) and PAL (b) in plasma and colonic mucosa during acetic acid (AA)‐induced ulcerative colitis. Animals were pretreated with various doses of DFAE (50, 100, and 200 mg/kg, *b.w*., *p.o*.), SULF (100 mg/kg, *b.w*., *p.o*.), or GA (50 mg/kg, *b.w*., *p.o*.) and distilled water. Rats were challenged with a single anal administration of AA (3%, v/v, 5 mL/kg, *b.w*.) or NaCl (0.9%, 5 mL/kg, *b.w*.) for 24 h. Data are expressed as means ± SD (*n* = 6) in each group. ***p < 0.001 compared to the control group; ^#^ *p* < 0.05, ^##^ *p* < 0.03, and ^*###*^ *p* < 0.001 compared to the acetic acid group.

The results of this study indicated that the intoxication with AA induced a significant (*p* < 0.05) increase in plasma and colonic mucosa C‐reactive protein content and alkaline phosphatase activity (Figure 6). The increase in these inflammatory markers compared with the control group was corrected with pretreatment with GA and SULF. Additionally, an interesting protection degree had been shown in the higher dose of DFAE recording the best anti‐inflammatory effect in a dose‐dependent manner.

### 3.9 Effect of DFAE on hepato‐renal dysfunction

Our results showed that the intrarectal administration of AA caused an increase in AST, ALT, creatinine, and urea (Table 5). However, groups pre‐treated with different doses of DFAE demonstrated significant protection against AA‐induced renal and hepatic dysfunction. Our results also show that GA and SULF, used as reference molecules, protect against hepato‐renal toxicity.

**TABLE 5:** Protective effects of Dill fruit aqueous extract (DFAE), sulfasalazine (SULF) and gallic acid (GA) on deregulation of renal and hepatic parameters following AA‐induced ulcerative colitis in rats. Animals were pretreated with various doses of DFAE (50, 100, and 200 mg/kg, *b.w., p.o*.), SULF (100 mg/kg, *b.w.*, *p.o*.) or GA (50 mg/kg, *b.w.*, *p.o*.) and distilled water. Rats were challenged with a single anal administration of AA (3%, v/v, 5 mL/kg, *b.w*.) or NaCl (0.9%, 5 mL/kg, *b.w*.) for 24 h.

Treatment	AST (UI/L)	ALT (UI/L)	Urea (mmol/L)	Creatinine (μmol/L)
Control	40 ± 1.36	24.16 ± 1.92	3.13 ± 0.09	80.32 ± 0.48
AA	56.17 ± 1.79*	65 ± 2.51*	8.16 ± 0.06*	120.76 ± 0.54*
AA + DFAE‐50	44 ± 2.39^#^	28.83 ± 1.92^#^	3.9 ± 0.28^#^	87.53 ± 3.46^#^
AA + DFAE‐100	42.83 ± 1.44^#^	26.67 ± 1.28^#^	3.88 ± 0.34^#^	85.02 ± 1.76^#^
AA + DFAE‐200	38 ± 1.61^#^	25,5 ± 1.52^#^	3.17 ± 0.06^#^	81.60 ± 2.18^#^
AA + SULF	35.67 ± 1.28^#^	26.17 ± 2.18^#^	3.41 ± 0.08^#^	81.74 ± 2.09^#^
AA + GA	37.67 ± 1.08^#^	25.33 ± 1.78^#^	3.32 ± 0.1^#^	81.96 ± 0.6^#^

*Note*: Data are expressed as means ± SD (*n* = 6).

* *p* < 0.05 compared with control group.

^#^ *p* < 0.05 compared with AA group.

### 3.10 Effect of DFAE on metabolic parameters and electrolytes

We have also demonstrated that acetic acid intoxication causes an alteration in lipid and glycemic balance. Pre‐treatment with DFAE, SULF, and GA significantly (*p* < 0.05) improved these metabolic disorders in a dose‐dependent manner (Table 6). The mineral metabolism parameters (Mg, Ca, and P) were also significantly restored by pretreatment with various doses of DFAE. Similar effects were observed for gallic acid and sulfasalazine used as reference molecules (Table 6).

**TABLE 6:** Effects of dill fruit aqueous extract (DFAE), sulfasalazine (SULF), and gallic acid (GA) on deregulation of metabolic parameters following AA‐induced ulcerative colitis in rats. Animals were pretreated with various doses of DFAE (50, 100, and 200 mg/kg, *b.w.*, *p.o*.), SULF (100 mg/kg, *b.w.*, *p.o*.), or GA (50 mg/kg, *b.w.*, *p.o*.) and distilled water. Rats were challenged with a single anal administration of AA (3%, v/v, 5 mL/kg, *b.w*.) or NaCl (0.9%, 5 mL/kg, *b.w*.) for 24 h.

Treatment	Glucose (mmol/L)	Cholesterol (mmol/L)	Triglycerides (mmol/L)	Ca (mmol/L)	Mg (mmol/L)	*p* (mmol/L)
Control	3.98 ± 0.03	0.98 ± 0.02	0.87 ± 0.05	1.34 ± 0.08	0.60 ± 0.03	1.18 ± 0.02
AA	7.58 ± 0.28*	2.12 ± 0.05*	2.13 ± 0.07*	2.93 ± 0.16*	1.25 ± 0.07*	2.98 ± 0.03*
AA + DFAE‐50	4.62 ± 0.08^#^	1.18 ± 0.03^#^	1.03 ± 0.15^#^	1.7 ± 0.17^#^	0.75 ± 0.01^#^	1.3 ± 0.01^#^
AA + DFAE‐100	4.56 ± 0.17^#^	1.11 ± 0.03^#^	0.81 ± 0.14^#^	1.64 ± 0.11^#^	0.65 ± 0.03^#^	1.27 ± 0.04^#^
AA + DFAE‐200	4.03 ± 0.04^#^	1.07 ± 0.09^#^	0.65 ± 0.07^#^	1.35 ± 0.01^#^	0.63 ± 0.04^#^	1.24 ± 0.01^#^
AA + SULF	4.28 ± 0.10^#^	1.05 ± 0.01^#^	0.66 ± 0.02^#^	1.45 ± 0.13^#^	0.76 ± 0.01^#^	1.25 ± 0.02^#^
AA + GA	4.26 ± 0.17^#^	1.13 ± 0.01^#^	0.67 ± 0.06^#^	1.46 ± 0.07^#^	0.74 ± 0.01^#^	1.26 ± 0.05^#^

*Note*: Data are expressed as mean ± SD (*n* = 6).

* *p* < 0.05 compared with control group.

^#^ *p* < 0.05 compared with AA group.

## 4 DISCUSSION

Ulcerative colitis patients turn to natural herbal products and functional foods, such as flavonoids, for treatment. In this study, we evaluated the phytochemical/antioxidant properties of *Anethum graveolens* L. fruit extract and its prophylactic action against AA‐induced ulcerative colitis in rats, as a model for inflammatory bowel disease.

Phytochemical analysis of dill fruit aqueous extract (DFAE) revealed significant levels of phenolic compounds. According to Paven's study, dill seeds contained 3.5 times more total polyphenols than the leaves (Paven et al., 2018). Interestingly, dill fruit contains high amounts of total fiber (soluble and insoluble), which may be beneficial for studying AA‐induced colitis models. However, the fiber‐containing diet resists digestion by intestinal enzymes and is more susceptible to fermentation in the colon, where it can be fermented to butyrate. In the years to come, the ability of fiber to maintain remission of ulcerative colitis is likely to come under closer scrutiny (Loy et al., 2024). Also, it may help minimize inflammation, modulate the immune response, restore the gut microbiome, and prevent cervical cancer in IBD, improving overall body health (Yusuf et al., 2022).

The main molecules detected in dill fruits by HPLC analysis are quercetin and resveratrol. Previous studies on the phenolic composition of dill fruits have shown considerable variation depending on plant type, seasonal stage, and climatic conditions (Balanescu et al., 2022; Manea et al., 2020; Paven et al., 2018). These molecules are responsible for DPPH^•^ radical scavenging activity, as revealed by the low IC_50_ value. The healing properties of dill are linked to its antioxidant and anti‐inflammatory properties, and the synergistic action of these bioactive compounds exerts numerous pharmacological effects (Manea et al., 2020). The development of new drugs based on dill to treat human diseases is due to its efficacy and safety.

Acetic acid‐induced experimental colitis in rats is a well‐known animal model for IBD research (Aleisa et al., 2014). Due to its simplicity, a high success rate and can be detected as early as 4 h due to obvious symptoms. Concentrations ranging from 3% to 8% induced colitis and showed morphological similarities with human ulcerative colitis (Jedidi et al., 2021; Owusu et al., 2020; Sammari et al., 2024). Acetic acid causes mild acute inflammation of the mucosa in the distal colon of rats.

Acute anal exposure to acetic acid (3% v/v, 5 mL/kg, *b.w*.) causes a series of reactions in rats, leading to the development of macroscopic lesions of the colonic mucosa with oxidative stress, hemorrhage, and congestion. The result is a state of oxidative stress, bleeding and congestion, edema, and even inflammation. We first showed that, like human ulcerative colitis, there is a morphological disorder associated with severe ulceration of the colonic mucosa and increased wet weight in the distal colon. Colon changes such as colonic mucosal inflammation, ulceration, and bleeding are also common in human inflammatory bowel disease (McDowell et al., 2024).

Ulcerative colitis is characterized by a thickening of the colonic wall, with an average thickness of 8 mm, compared with the average thickness of a normal colonic wall of 2 to 3 mm. In histopathological studies, several microscopic aspects were observed, including architectural abnormalities (branching or atrophy of crypts, glandular distortion, crypt abscesses, etc.) and lympho‐plasmocytic infiltration of the chorion. In the present study, intrarectal administration of AA induced significant histopathological changes in colonic thickening, hyperemia, caliciform cell hyperplasia, and inflammatory infiltrates. The positive effect of pretreatment with DFAE was characterized by interesting corrections in tissue healing, inflammatory biomarkers, and histopathological scores indicating improvements in remission rates. Importantly, histopathological images in the groups pretreated with DFAE, SULF, and GA revealed a significant improvement in the epithelial barrier in a dose‐dependent manner. These results are similar to those reported by other investigators (Jedidi et al., 2021; Sammari et al., 2024). This colonic protection is thought to be due in part to the richness of DFAE's in tannins and polyphenols, particularly flavonoids, which are thought to have anti‐ulcer and gastroprotective effects. According to our results, dill fruit is rich in fiber, which improves digestion in the large intestine, characterized by a high presence of bacteria.

The intrarectal administration of acetic acid in animals causes an imbalance between oxidants and antioxidants. Colitis models generally show an increase in oxidative stress and a decrease in antioxidant activity (Sahoo et al., 2023). This experimental model is associated with inflammatory responses and oxidative reactions that mimic the pathogenesis of human inflammatory bowel disease (Wu et al., 2020). In our investigation, we demonstrated that anal administration of acetic acid induces a state of oxidative stress in the colonic mucosa, manifested by an increase in lipid peroxides and a decrease in the activity of antioxidant enzymes such as SOD, CAT, and GPx, as well as deleterious effects on nonenzymatic antioxidants such as sulfhydryl groups. These results corroborate research by Satyanarayana et al. who confirmed the role of reactive oxygen species in colon pathogenesis (Satyanarayana et al., 2004). Pre‐treatment with DFAE for 10 days restored deregulation of the colonic mucosa thanks to flavonoids with antioxidant activity in a dose‐dependent manner. DFAE's natural active ingredients prevent AA‐induced inflammation through its anti‐inflammatory and analgesic effects (Naseri et al., 2012).

Pretreatment with SULF and GA resulted in a significant improvement in the unbalanced oxidative status following AA intoxication. Sulfasalazine, a drug consisting of 5‐ASA conjugated to sulfapyridine by colonic bacteria, has an anti‐inflammatory effect (Lu & Zhao, 2020). 5‐aminosalicylic acid (5‐ASA), an NF‐кB antagonist, suppresses intestinal inflammation by blocking cyclo‐oxygenase and inhibiting prostaglandin production (Loddo & Romano, 2015; Yang et al., 2016). It also inhibits the NF‐κB signaling pathway, reducing the production of pro‐inflammatory cytokines (Hauso et al., 2015). Its long‐term use, even at low doses, can cause serious side effects, including cardiovascular disease, cataracts, infections, and metabolic syndrome (Chan & Ng, 2017). Gallic acid (3,4,5‐trihydroxybenzoic acid) is a phenolic acid found in many foods, including fruits and vegetables (Amakura et al., 2000). This compound has been shown to possess antioxidant and anti‐inflammatory properties (Choubey et al., 2015), as well as anticancer (Verma et al., 2013) and cytoprotective effects (Mansouri et al., 2013). Recently, it has been shown its ability to attenuate inflammation by suppressing pro‐inflammatory cytokines and inflammatory mediators such as iNOS and COX^−2^ (Pandurangan et al., 2015). The improvement in biological antioxidant status is due to the activation of cytoprotection, the reduction of prostaglandin metabolism in the mucosa, and the reduction of vascular permeability.

Fe^2+^ and Fe^3+^ catalyzed the dismutation of H_2_O_2_ via the Fenton reaction producing OH‐ and superoxide, respectively (Abe et al., 2022). Pretreatment with DFAE, SULF, and GA using phenolic compounds such as polyphenols and tannins can reduce oxidative stress and neutralize OH‐hydroxyl radicals. A significant correction of Ca^2+^ homeostasis has also been demonstrated under the action of tannins, which leads to muscle relaxation.

Our study demonstrated that intrarectal administration of acetic acid disrupts inflammatory markers such as CRP and ALP. Alkaline phosphatase (ALP) is a group of isozymes located in the outer layer of cell membranes, whose essential cofactors are zinc and magnesium. It is present in the cytosol of hepatocytes and catalyzes the hydrolysis of organic phosphate esters in the extracellular space (Lowe et al., 2023). In our investigation, we reported that pre‐treatment with DFAE can significantly correct plasma levels of CRP and ALP. Indeed, dill has antinociceptive properties; it can be used as an analgesic and relieve inflammatory pain by inhibiting inflammatory mediators (Rezaee Asl et al., 2013). A pilot study showed a decrease in serum levels of tumor necrosis factor‐alpha TNF‐α, interleukin IL‐6 and IL‐8 in ulcerative colitis patients in remission who received sprouted barley (Faghfoori et al., 2011). In 2014, the same researchers conducted a follow‐up study to examine how sprouted barley supplements can effectively reduce inflammation. The results showed that serum C‐reactive protein (CRP) levels and clinical symptoms in patients with ulcerative colitis were decreased significantly in the fiber‐enriched and fiber‐reinforced group (Faghfoori et al., 2014).

On the other hand, rectal administration of acetic acid has been shown to destroy lipid status and significantly increase blood glucose levels. Hyperlipidemia is a metabolic disorder characterized by increased levels of lipoproteins and cholesterol in the blood (Pandit et al., 2015). Pre‐treatment with DFAE corrected these metabolic alterations, as dill has antidiabetic (Goodarzi et al., 2016) and antihyperlipidemic effects (Sahib et al., 2012).

The colon is involved in the absorption of water and electrolytes, as well as in the formation, movement, and storage of unabsorbed material such as fecal matter. Mineral elements play an essential role in human health (Kumar Sahu et al., 2022). We demonstrated that anal administration of AA induced an electrolyte imbalance compared with groups pre‐treated with DFAE, SULF, and GA in a dose‐dependent manner. The correction of this alteration by dill fruits is due to their high mineral content (Isopencu & Ferdeş, 2012). This is consistent with the study by Haidari et al. which showed that dill fruits induced a significant, dose‐dependent repair of serum levels of glycemic and lipid parameters, certain antioxidants, inflammatory markers, and gastrointestinal symptoms in patients with type 2 diabetes (Haidari et al., 2020).

Our study revealed macroscopic, microscopic, and biochemical improvements from protective treatment with this natural herb. Compared with placebo, dill significantly increased maintenance of response to treatment. Optimal treatment strategies include reducing inflammatory lesions and the expression of pro‐inflammatory molecules. Key treatments include disease education and dietary changes to improve quality of life. This work can be reinforced by biomedical technology, which enables us to study various human diseases on a genome‐wide scale. Hence, “omics” testing of molecular and cellular data from biomaterials (tissue, blood, urine, and stool) could contribute to our understanding of ulcerative colitis pathogenesis and the promotion of new drugs for its patients by targeting essential networks of this interactome.

## 5 CONCLUSION

Dill fruit showed a powerful cytoprotective effect against ulcerative colitis due to its antioxidant and anti‐inflammatory activities. The high phenolic compound and dietary fiber content of DFAE prevented acetic acid‐induced experimental colitis while helping the colonic mucosa maintain its structure, minimizing oxidative stress, inflammation, and metabolic biomarker deregulations. It can be considered a natural remedy against gastrointestinal diseases as a protective measure.

## AUTHOR CONTRIBUTIONS

Chirine Brinsi, Yassin Khadhraoui, Mariem Mhimdi, Slimen Selmi, Houcem Sammari, Saber Jedidi, and Houcine Selmi conducted the experimental study, data acquisition, analysis, and manuscript preparation. Chirine Brinsi conducted the manuscript editing, literature research, and data analysis. Chirine Brinsi and Hichem Sebai conducted the study concept and design, manuscript preparation, and review. All authors read and approved the final manuscript.

## FUNDING INFORMATION

The author(s) received no financial support for the research, authorship, and/or publication of this article.

## CONFLICT OF INTEREST STATEMENT

The authors declare that no conflicts of interest exist.

## ETHICS STATEMENT

All maintenance and sacrifice procedures were used following the local ethics committee of Tunis University regarding the use and care of animals and by the NIH recommendation. The protocol was approved by the “Comité d'Ethique Bio‐medicale (CEBM)” (JORT472001) of the “Institut Pasteur de Tunis.”

## Data Availability

The data that support the findings of this study are available on request from the corresponding author. The data are not publicly available due to privacy or ethical restrictions.
